# Akt-ing Up Just About Everywhere: Compartment-Specific Akt Activation and Function in Receptor Tyrosine Kinase Signaling

**DOI:** 10.3389/fcell.2019.00070

**Published:** 2019-05-03

**Authors:** Michael G. Sugiyama, Gregory D. Fairn, Costin N. Antonescu

**Affiliations:** ^1^Department of Chemistry and Biology, Ryerson University, Toronto, ON, Canada; ^2^Keenan Research Centre for Biomedical Science, St. Michael’s Hospital, Toronto, ON, Canada; ^3^Department of Surgery, University of Toronto, Toronto, ON, Canada

**Keywords:** receptor tyrosine kinase, endocytosis, plasma membrane, endosome, lysosome, nucleus, phosphatidylinositol-3, 4, 5-trisphosphate, phosphatidylinositol-3-kinase

## Abstract

The serine/threonine kinase Akt is a master regulator of many diverse cellular functions, including survival, growth, metabolism, migration, and differentiation. Receptor tyrosine kinases are critical regulators of Akt, as a result of activation of phosphatidylinositol-3-kinase (PI3K) signaling leading to Akt activation upon receptor stimulation. The signaling axis formed by receptor tyrosine kinases, PI3K and Akt, as well as the vast range of downstream substrates is thus central to control of cell physiology in many different contexts and tissues. This axis must be tightly regulated, as disruption of PI3K-Akt signaling underlies the pathology of many diseases such as cancer and diabetes. This sophisticated regulation of PI3K-Akt signaling is due in part to the spatial and temporal compartmentalization of Akt activation and function, including in specific nanoscale domains of the plasma membrane as well as in specific intracellular membrane compartments. Here, we review the evidence for localized activation of PI3K-Akt signaling by receptor tyrosine kinases in various specific cellular compartments, as well as that of compartment-specific functions of Akt leading to control of several fundamental cellular processes. This spatial and temporal control of Akt activation and function occurs by a large number of parallel molecular mechanisms that are central to regulation of cell physiology.

## Introduction

Signaling by the phosphatidylinositol-3-kinase (PI3K)-Akt pathway is a central regulator of cell growth, metabolism, and survival (Fruman et al., 2017; Manning and Toker, 2017). PI3K-Akt signaling is involved in a wide range of physiological processes in many different cells and tissues, and at various stages of development including homeostasis of adult tissues. Disruptions of PI3K-Akt signaling also contribute to disease, such as the insulin resistance and type II diabetes that results from impaired PI3K-Akt signal transduction in insulin-responsive tissues (Boucher et al., 2014; Manning and Toker, 2017). In contrast, inappropriate amplification of PI3K-Akt signaling is present in many different types of cancer cells thereby driving cell growth and tumor progression (Cheng et al., 2005; Carracedo and Pandolfi, 2008; Liu et al., 2009; Fruman and Rommel, 2014; Thorpe et al., 2015; Fruman et al., 2017; Manning and Toker, 2017).

PI3K-Akt signaling occurs following a wide range of cues emanating from the plasma membrane, such as receptor tyrosine kinases, G-protein coupled receptors and immune receptors (Fruman et al., 2017; Manning and Toker, 2017). Activation of these receptors, typically but not exclusively resulting from ligand binding, leads to signals that classically activate class I PI3K, leading to the production of phosphatidylinositol-3,4,5-trisphosphate (PIP_3_) from phosphatidylinositol-4,5-bisphosphate (PI45P_2_). In addition, receptor signaling can lead to the production of phosphatidylinositol-3,4-bisphosphate (PI34P_2_), either by dephosphorylation of PIP_3_ by 5-phosphatases or by activation of class II PI3Ks that phosphorylate phosphatidylinositol-4-phosphate (PI4P). PIP_3_ and PI34P_2_ trigger membrane recruitment and potentiate signals that lead to the activation of Akt. In turn, Akt exerts control of >100 substrates distributed throughout the cell including the plasma membrane, various endomembrane compartments, the mitochondria, cytosol, and the nucleus (Wang and Brattain, 2006; Santi and Lee, 2010; Ebner et al., 2017a), thus directing multiple facets of cell physiology.

A central question in the regulation of receptor signaling is the control of these signals by their spatiotemporal organization, such as afforded by the endomembrane system. Many receptor complexes initiate signaling at the plasma membrane and subsequently undergo endocytosis upon ligand binding leading to the transit of active receptor signaling complexes through various endomembranes, which depending on the membrane traffic itinerary of each receptor can include early, recycling and late endosomes (Scita and Di Fiore, 2010; Goh and Sorkin, 2013; Barbieri et al., 2016). This endocytic movement has led to the central hypothesis that the plasma membrane and various internal membrane compartments represent distinct signaling environments, such that activated receptors may trigger unique signals from each membrane locale. Moreover, the plasma membrane, and by extension various endomembrane compartments are non-homogenous lipid bilayers, comprised of various nanodomains defined by the presence of unique proteins such as clathrin, caveolin, flotillin and tetraspanins, as well as actin-dependent nanodomains, each of which also represent unique signaling nano-environments for activated receptors (Delos Santos et al., 2015; Lu and Fairn, 2018).

Distinct signaling environments, ranging from nanoscale domains at the plasma membrane to microscale endomembrane compartments represent opportunities for distinct activation, regulation or functional outcome of PI3K-Akt signaling. In this review, we first examine the identity of the molecular players that trigger the initial activation of and/or regulate PI3K-Akt signaling by cues that initiate at the cell surface. Although Akt activation also occurs in response to other intracellular cues, such as in response to DNA damage (Liu et al., 2014), we focus here on signals triggered at the plasma membrane by receptor tyrosine kinases (RTKs). We direct the reader to several excellent recent reviews that examine the spatiotemporal organization of signaling by GPCRs and other receptors (Kholodenko et al., 2010; Subramanyam and Colecraft, 2015; Gahbauer and Böckmann, 2016; Calebiro and Sungkaworn, 2018). We examine the evidence for specific localization of PI3K-Akt signals to various subcellular compartments, including nanoscale domains of the plasma membrane and various endomembrane compartments. Subsequently, we examine the evidence that the spatiotemporal organization of PI3K-Akt signaling within these various compartments may result in distinct outcomes of Akt signaling at each locale.

## Molecular Mechanisms of PI3K-AKt Signal Activation

Receptor tyrosine kinases are a family of 58 human proteins that are critical for a wide range of physiological processes, from development to maintenance of tissue homeostasis in adults (Lemmon and Schlessinger, 2010; Lemmon et al., 2014). RTK activation can trigger signals that promote cell growth, proliferation, survival, migration or differentiation, and these outcomes depend on the specific ligand, receptor, and cellular context. RTKs for the most part bind extracellular ligands, which then leads to activation of intrinsic kinase domains that then relay the signal from ligand binding to intracellular signaling networks (Yarden, 2001; Lemmon and Schlessinger, 2010). RTK activation typically occurs either by ligand binding to a constitutive receptor dimer (as is the case for insulin receptor) (McKern et al., 2006; Siddle, 2011), or by stabilization of a receptor dimer (as is the case for the epidermal growth factor receptor, EGFR) (Alvarado et al., 2010).

For many RTKs, activation of their intrinsic kinase domain results in phosphorylation of multiple tyrosine residues within cytosolic motifs of the receptor itself, which in turn serve as ligands to recruit signaling adaptors or enzymes harboring phospho-tyrosine binding (PTB) or Src-homology 2 (SH2) domains. This, in turn, leads to activation of a vast network of signals, which has been extensively reviewed elsewhere (Yarden and Shilo, 2007; Lemmon and Schlessinger, 2010; Wagner et al., 2013; Wee and Wang, 2017). Here, we focus on signals that lead to activation of PI3K, leading in turn to the production of specific phosphoinositides, such as PIP_3_ and PI34P_2_, and subsequently Akt activation. To this end, we discuss PI3K enzymes, how these are activated by direct binding to RTKs or to scaffolding proteins, and how this leads to the activation of Akt. We also consider negative regulation by relevant lipid and protein phosphatases.

### PI3K Isoforms

Phosphatidylinositol-3-kinase enzymes are classified into three classes (I–III). Class I PI3K are comprised of one of several p110 catalytic subunits and one regulatory subunit of variable size (Jean and Kiger, 2014; Thorpe et al., 2015). RTKs primarily activate a subset within this group, Class 1A PI3K, which are comprised of a heterodimer of one p110α, β, or δ catalytic subunit and one p85α (or splice variants p50α and p55α), p85β or other regulatory subunits. Class 1B PI3Ks are comprised of the p110γ catalytic subunit and the p101 and p87 regulatory subunits and are largely activated by GPCR signaling (Vadas et al., 2013), and thus will not be examined here as we focus on RTK signaling. For Class IA PI3Ks, the interaction of regulatory and catalytic subunits is constitutive and in the absence of signals serves to suppress the p110 subunits (Miled et al., 2007; Vadas et al., 2011). Binding of the regulatory subunit to specific motifs harboring phosphorylated tyrosines (e.g., on RTKs or scaffolding proteins, see below) via its SH2 domains relieves the inhibition on p110 subunits. Specific regulatory subunits have additional domains that expand the mechanisms of activation, such as the p110 subunits that can also be activated by binding to Ras via N-terminal Ras-activating domains (Vanhaesebroeck et al., 2010). The activation of Class IA PI3Ks leads to production of PIP_3_ from PI45P_2_.

Class II PI3Ks are comprised of three isoforms in humans, PI3KC2α, β, and γ (Falasca and Maffucci, 2007; Posor et al., 2013, 2014; Marat et al., 2017). These isoforms are each comprised of C2 and PX domains that mediate binding to lipids, especially PI45P_2_ (Liu et al., 2006; Stahelin et al., 2006; Wang et al., 2018), and a kinase domain that catalyzes the formation of PI34P_2_ from PI4P, as well as phosphatidylinositol-3-phosphate (PI3P) from phosphatidylinositol (Falasca and Maffucci, 2007; Posor et al., 2013). Additional protein interaction domains and activities are present in each isoform, such as binding to clathrin for PI3KC2α and β (Domin et al., 2000; Gaidarov et al., 2005; Posor et al., 2013). The regulation of Class II PI3Ks is less well understood. Class II PI3Ks may constitutively associate with membranes and require additional activation signals such as the conformational change in PI3KC2α induced by binding to specific proteins and PI45P_2_ (Wang et al., 2018).

Class III PI3K has a sole member, Vps34, which functions at the early endosome to produce PI3P from PI (Schu et al., 1993; Kim et al., 2013; Backer, 2016). Vps34 and PI3P are essential for membrane traffic from the plasma membrane to early endosomes and also regulate a number of sorting phenomena, including assembly of the retromer cargo retrieval complex (Herman and Emr, 1990). As PI3P produced by Vps34 does not appear to contribute to activation of Akt signaling directly, we here focus on Class I and II PI3Ks and discuss Vps34 only in the context of its requirement for membrane traffic regulation of Akt signaling.

#### PI3K Activation by RTKs

As a result of decades of intensive research, there is considerable insight into the mechanism of activation of Class IA PI3K by RTKs. Some receptor tyrosine kinases can directly bind and thus activate PI3Ks, while others require a scaffolding or binding protein. Here, we illustrate the latter with EGFR and the former mechanism with ErbB3.

In the case of EGFR, binding to ligands leads to activation of the kinase domain, which in turn, leads to the phosphorylation of a number of residues on the C-terminal tail of the receptor (Bessman et al., 2014; Lemmon et al., 2014; Freed et al., 2017). The phosphorylation of Y1068 is essential for binding of Grb2 via its SH2 domain, which then recruits Grb2-associated binder1 (Gab1) via an SH3-proline rich domain interaction (Lock et al., 2000). The phosphorylation of Gab1 on Y447, Y472, and Y589 leads to recruitment of Class IA PI3Ks, and production of PIP_3_ leading to Akt activation (Holgado-Madruga et al., 1996; Mattoon et al., 2004; Kiyatkin et al., 2006). Gab1 possesses a PH domain that binds PIP_3_, and as Gab1 membrane binding contributes to PI3K activation, this mechanism of activation of PI3K is subject to positive feedback regulation (Rodrigues et al., 2000). Other RTKs such as MET use a similar mechanism of activation, but MET can directly bind Gab1 via a Gab1-binding module found in the cytosolic portion of this receptor (Schaeper et al., 2007). Gab1 is related to other scaffolding or docking proteins that function to control PI3K activation by RTKs, including insulin-receptor substrate (IRS) and fibroblast growth factor receptor substrate 2 (FRS2), which either recruit class IA PI3K directly (Brummer et al., 2010; Shaw, 2011; Boucher et al., 2014) or via Gab1 recruitment upon activation of specific RTKs (Kouhara et al., 1997; Melillo et al., 2001; Schlessinger, 2004; Degoutin et al., 2007; Gotoh, 2008).

In contrast to adaptor-mediated recruitment, other RTKs can directly bind PI3K. Upon binding its ligand, such as neuregulin (Carraway et al., 1997), and phosphorylation, ErbB3 interacts directly with class IA PI3K leading to PI3K activation (Hellyer et al., 1998; Baselga and Swain, 2009; Smirnova et al., 2012). Given the distinction of direct receptor binding *versus* adaptor-dependent PI3K-Akt signal activation by various RTKs, spatial-temporal differences and strength of the signal may occur; however, this remains to be explicitly examined.

Class II PI3Ks can also be activated downstream of RTK activation. PI3KC2α and β are recruited to EGF and platelet-derived growth factor receptor (PDGFR) signaling complexes (Arcaro et al., 2000), via recruitment to adaptor protein complexes that include Grb2 (Wheeler and Domin, 2001; Katso et al., 2006). The detailed mechanisms for this class of PI3K remain much less well understood than class I PI3K, and additional insight into the regulation of class II PI3K would be very informative.

### Akt Activation by RTK Signaling

The production of either PI34P_2_ or PIP_3_ is essential for the recruitment and activation of Akt by RTKs. There are three isoforms of Akt (1–3), each comprised of an N-terminal pleckstrin homology (PH) domain, an internal kinase domain, and a C-terminal regulatory domain. The isolated PH domain of each isoform of Akt is able to bind either PI34P_2_ or PIP_3_ with similar affinity, but in the context of the full-length protein, Akt1 and 3 preferentially bind PIP_3_ while Akt2 preferentially binds PI34P_2_ (Liu et al., 2018). In each case, binding to either PI34P_2_ or PIP_3_ elicits recruitment of Akt to the membrane, in parallel to similar membrane recruitment by these lipids of 3-phosphoinositide-dependent kinase 1 (PDK1) via its PH domain. PDK1 phosphorylates Akt on T308, which together with membrane binding leads to a substantial increase in Akt activity (Stephens et al., 1998; Scheid et al., 2002; Higuchi et al., 2008). Phosphorylation of Akt on S473 further enhances Akt activity, which in the context of RTK signaling is elicited by the mechanistic target of rapamycin complex 2 (mTORC2) (Sarbassov et al., 2005; Oh and Jacinto, 2011; Gaubitz et al., 2016). Alternatively, phosphorylation can also be mediated by DNA-PK in other contexts (Feng et al., 2004; Bozulic et al., 2008; Szymonowicz et al., 2018). Regardless, the dually phosphorylated Akt has a substantial increase in activity (Hart and Vogt, 2011).

While membrane binding and phosphorylation on T308 and S473 represent the canonical activation of Akt by RTKs, there is an increasing appreciation of many different modifications and regulators of Akt (Risso et al., 2015). Notably, K63-ubiquitinylation of Akt on K8 and K14, mediated by TRAF6 upon IGF1 stimulation (Yang et al., 2009) or Skp2 SCF upon EGF stimulation (Chan et al., 2012) is required for Akt activation, membrane recruitment and substrate phosphorylation, a modification that is negatively regulated by the deubiquitinase CYLD (Yang et al., 2013). This K63-ubiquitinylation does not appear to control Akt degradation. Further adding to the complexity of regulation of Akt activation by post-translational modification, Akt methylation on K64 by SETDB1 promotes the binding of JMJD2A, which functions as an adaptor to recuit TRAF6 or the Skp2 SCF and K63-ubiquitinylation of Akt, required for Akt activation (Wang et al., 2019). While mechanisms such as K63-mediated ubiquitinylation control Akt activation, much remains to be determined about how these mechanisms are regulated, and how these impact Akt cellular localization.

All three isoforms of Akt follow this activation mechanism and share some overlapping substrates. However, there are substrates of Akt that are isoform-specific, and in many contexts, Akt isoforms are non-redundant with distinct roles in cellular and systemic physiology (Stambolic and Woodgett, 2006; Gonzalez and McGraw, 2009b; Schultze et al., 2011; Roy et al., 2017). For example, Akt1 but not Akt2 phosphorylates palladin, an actin-bundling protein (Chin and Toker, 2010). This may contribute to the distinct ability of Akt1 to promote breast tumor initiation and impair invasion and migration (Hutchinson et al., 2004; Irie et al., 2005), while Akt2 enhances the invasive and metastatic capabilities of breast tumors (Arboleda et al., 2003; Irie et al., 2005).

### Attenuation of PI3K-Akt Signaling Downstream of RTKs

Given the impact on various aspects of cell physiology, mitogenic signaling emanating from RTKs and propagated through PI3K-Akt signaling is subject to negative regulation at various levels. A large number of phosphatases negatively regulate phosphorylation of RTKs and their adaptors and scaffold/docking proteins (Lemmon and Schlessinger, 2010; Yao et al., 2017; Neben et al., 2019). RTKs are also subject to negative regulation by degradation secondary to internalization. Examination of this aspect of RTK signaling is beyond the scope of this review, but we direct the reader to several excellent comprehensive reviews on this subject (Roepstorff et al., 2008; Sorkin and Goh, 2009; Sorkin and von Zastrow, 2009; Hurley, 2010; Caldieri et al., 2018; Critchley et al., 2018).

Several lipid and protein phosphatases regulate these signals directly at the level of PI3K-Akt. PTEN is a lipid phosphatase that negatively regulates PI3K-Akt signaling by dephosphorylation of PIP_3_ to produce PI45P_2_ (Lee et al., 2018), and also negatively regulates PI34P_2_ (Malek et al., 2017). Given its central role in negative regulation of PI3K-Akt signaling, PTEN is a potent tumor suppressor, and disruptions of PTEN actively promote tumor growth and progression (Lee et al., 2018). In addition, SH2-domain containing inositol phosphatase 2 (SHIP2, also known as INPP1L) dephosphorylates the 5-position of PIP_3_, leading to the production of PI34P_2_ (Goulden et al., 2018; Liu et al., 2018). While regarded in some ways as a negative regulator of PI3K-Akt signaling by catalysis of turnover of PIP_3_, that SHIP2 leads to the production of PI34P_2_ may potentiate the activation of specific isoforms of Akt. Indeed SHIP2 is responsible for the production of PI34P_2_ that selectively activates Akt2 (Liu et al., 2018). An additional phosphatase, INPP4B, has recently emerged as a negative regulator of PIP_3_ and PI34P_2_ (Kofuji et al., 2015), yet other studies have noted that INPP4B promotes Akt signaling by relieving negative regulation of Class I PI3K (Reed and Shokat, 2017). Hence, while PTEN and INPP4B are potent suppressors of PI3K-Akt signaling, SHIP2 has a more complex role in the regulation of this pathway.

Several phosphatases act to directly regulate phosphorylation of Akt. Protein phosphatase 2A (PP2A) is a well-established negative regulator of Akt that elicits Akt dephosphorylation, in particular on the T308 site (Rodgers et al., 2011; Seshacharyulu et al., 2013). In addition, PH domain leucine-rich repeat protein phosphatase (PHLPP) 1 and 2 are two phosphatases that act selectively on the S473 site (Gao et al., 2005). Interestingly, these two PHLPP isoforms exhibit specificity for different Akt isoforms, such that PHLPP1 regulates signaling by Akt2 and PHLPP2 regulates signaling by Akt3 (Brognard et al., 2007).

With this framework of activators and negative regulators of PI3K-Akt signaling, we next discuss the critical contributions of spatiotemporal activation of Akt signals at different scales: within nanodomains at the plasma membrane, and within endomembrane compartments. Subsequently, we examine how Akt functionally controls cell and systemic physiology, with a focus on compartment-specific activation and functions of Akt.

## Localization of PI3K-AKt Signaling Within Plasma Membrane Nanodomains

The initiation of signaling at the plasma membrane involves the spatiotemporal organization of receptors and cytoplasmic proteins that transduce extracellular signals to the appropriate intracellular destination. Recent technological advances, particularly in the field of live-cell fluorescence microscopy, have revealed that signaling receptors are heterogeneously distributed in the plasma membrane, as a result of enrichment in distinct plasma membrane nanodomains. These nanodomains vary in lifetime and composition and include cholesterol-rich structures (caveolae and flotillin), clathrin structures, tetraspanin-enriched nanodomains, dorsal actin ruffles, and Ras nanoclusters. From this vantage, these nanodomains serve to compartmentalize signaling complexes into transient signaling hotspots on the plasma membrane. In the case of caveola and clathrin, which are capable of forming *bona fide* endocytic vesicles, endocytosis of receptor/nanodomain complexes might also serve as a checkpoint for the redistribution of active signaling complexes to distinct subcellular locales or termination of the signal through degradative pathways. The following examines the evidence for localized PI3K-Akt signaling within specific plasma membrane nanodomains.

### Clathrin

Cells have adopted several unique mechanisms for the internalization of extracellular material, membrane proteins, lipids, and solutes. Perhaps the best-described mechanism involves the formation of clathrin-coated pits (CCPs) at the plasma membrane and subsequent clathrin-mediated endocytosis (CME). CCPs initiate by the recruitment of the clathrin adaptor protein complex 2 (AP2), to the plasma membrane by recognition of internalization motifs on cargo proteins destined for CME and by binding PI45P_2_ (Schmid and McMahon, 2007; Mettlen et al., 2009, 2018; McMahon and Boucrot, 2011; Taylor et al., 2011; Cocucci et al., 2012; Kadlecova et al., 2017). This is followed by the assembly of other components the clathrin coat, which in addition to clathrin includes accessory proteins, eventually leading to scission from the membrane by the GTPase dynamin. Following this internalization, nascent vesicles undergo uncoating, followed by membrane traffic and sorting. CME has been described in the context of cell signaling as an essential regulator of EGFR signaling dynamics, whereby EGF (ligand) stimulation of EGFR leads to internalization of the ligand/receptor complex (Sorkin and Goh, 2009; Goh and Sorkin, 2013; Schmid, 2017; Critchley et al., 2018). Internalized ligand/receptor complexes are delivered to the endosomal system, which can lead to ubiquitin-dependent lysosomal degradation or recycling back to the plasma membrane. Thus, depending on the cellular context, RTK internalization by CME can attenuate signaling through receptor internalization, lead to degradation, or prolong signaling by receptor recycling.

Beyond their role in endocytosis, recent evidence suggests that a subset of plasma membrane CCPs may also represent unique clathrin nanodomains that directly influence Akt signaling by orchestrating the assembly of transient receptor signaling complexes on the plasma membrane. This previously unrecognized role of clathrin nanodomains as signaling scaffolds suggests another critical level of control over receptor signaling. EGF treatment of ARPE-19 cells leads to the accumulation of EGF and phosphorylated Gab1 (pY627), the most receptor-proximal upstream activator of PI3K/Akt, in clathrin structures on the plasma membrane (Garay et al., 2015; Lucarelli et al., 2016, 2017). Perturbation of CCP formation, but not receptor endocytosis, attenuates Gab1 (pY307 and pY627) and Akt (pT308 and pS473) phosphorylation (Garay et al., 2015), supporting a role for some clathrin structures as signaling nanodomains required for PI3K-Akt signaling. Interestingly, the engineered expression of ErbB2 in ARPE-19 cells, which normally express little ErbB2, rescues the inhibitory effects of clathrin perturbation on Akt (pS473) phosphorylation (Garay et al., 2015).

In addition, phosphorylated Akt and PTEN preferentially localize to short-lived CCPs in MCF10A breast epithelial cells (Rosselli-Murai et al., 2018). Deletion of PTEN in these cells or addition of supplemental PIP_3_ enhanced the initiation of short-lived CCPs. These effects were mirrored in MDA-MB-231 and SUM149PT triple negative breast cancer cells, which lack ErbB2 and functional PTEN, respectively. Together, these studies suggest that a distinct subpopulation of clathrin structures at the plasma membrane form nanodomains required for PI3K-Akt activation in cells lacking ErbB2. In contrast, co-expression of ErbB2 leads to EGFR-dependent Akt activation that is clathrin-independent (Garay et al., 2015). Furthermore, PI3K/Akt signaling at clathrin nanodomains is directly influenced by the phosphatase activity of PTEN, through the control of PIP_3_ abundance on the plasma membrane (Rosselli-Murai et al., 2018). Collectively, these studies support the notion that a subset of clathrin structures function as signaling nanodomains at the cell surface, as has been proposed for certain aspects of GPCR signaling (Eichel et al., 2016, 2018; Eichel and von Zastrow, 2018).

Components of the PI3K/Akt signaling pathway impinge on different stages of CCP formation and play a reciprocal role in regulating CME. Akt activity positively regulates CME through a mechanism that leads to dephosphorylation and thus activation of dynamin-1 (pS774) (Reis et al., 2015). Collectively, these studies establish the existence of a reciprocal regulation network in which plasma membrane clathrin nanodomains directly facilitate Akt activation at the plasma membrane, followed by modulation of ligand/receptor complex traffic after CME. In turn, multiple levels of signaling converge to control CME.

### Caveolin and Flotillin Membrane Nanodomains

Caveolae are 50–100 nm bulb-shaped invaginations on the plasma membrane that are primarily composed of oligomers of the integral membrane protein caveolin-1, and the cavin proteins, which are essential for caveolae formation (Hill et al., 2008). Caveolae are typically thought of as a type of membrane rafts. This is due to the ability of caveolins to bind cholesterol, the sensitivity of caveolae to disruption of membrane cholesterol, and the low buoyant density of isolated caveolae (Smart et al., 1995). Insights into the role of caveolae in PI3K/Akt signaling has mostly been inferred through rather harsh disruption of the cell surface by cholesterol depletion or overexpression of caveolin-1 in cell lines without endogenous caveolin-1 expression (Parpal et al., 2001; Fiucci et al., 2002) but given the limitations of these approaches, the interpretation of such results should be taken with caution (Zhuang et al., 2002). Furthermore, caveolin-1 knockout mice are viable suggesting that essential growth factor signaling remains intact (Drab et al., 2001; Parton, 2001). While silencing of caveolin-1 has been reported to enhance Akt activity in endothelial cells (Gonzalez et al., 2004), the limitations of methods used to alter caveola have contributed to the inconsistencies in the literature concerning the effects of caveolin-1 on PI3K/Akt signaling, and thus results should be interpreted with caution.

Many studies revealed interaction of receptor tyrosine kinases with caveolin proteins or incorporation of receptors within caveolae (Delos Santos et al., 2015). The impact of this nanoscale compartmentalization is complex and in some cases impacts regulation at the level of the receptors themselves, thus broadly impacting multiple signaling pathways (Yamamoto et al., 1998; Nystrom et al., 1999; Baumann et al., 2000; Vainio et al., 2002; Cohen et al., 2003a,b, 2004; Foti et al., 2007; Wang et al., 2011; Bridges et al., 2012; Delos Santos et al., 2015; Yamaguchi et al., 2016; Lu and Fairn, 2018). In addition, while there is little direct evidence that EGFR is detected within caveolae (Delos Santos et al., 2015) interactions of EGFR within caveolin-1, perhaps in the context of non-caveolar assemblies of caveolin-1 proteins (Head and Insel, 2007; Lajoie et al., 2007; Nassar et al., 2015; Khater et al., 2018) negatively regulate EGFR (Couet et al., 1997; Engelman et al., 1998; Park et al., 2000; Williams et al., 2004; Lajoie et al., 2007; Lambert et al., 2008). Indeed, caveolin proteins harbor a caveolin-scaffolding domain (CSD, amino acids 82-101) that allows interaction with many different proteins (Jung et al., 2018), suggesting a mechanisms for how protein-protein interactions involving caveolins may control RTK signaling to PI3K-Akt.

While these studies establish several different possible modalities of control of receptor tyrosine kinase signaling by caveolins and caveolae at the level of the receptors, there is also evidence of enrichment and control of PI3K/Akt signaling intermediates in these structures. Both PI45P_2_ and PIP_3_ are detected in membrane nanodomains (Wang and Richards, 2012), and insulin-like growth factor (IGF1) stimulation triggers PIP_3_ partitioning into membrane nanodomains that are sensitive to cholesterol perturbation (Lasserre et al., 2008). While these studies establish the non-homogenous partitioning of PIP_3_ in the plasma membrane, it is not clear how these lipid nanoclusters relate to caveolae. Caveola or caveolin proteins may also regulate Akt activity through interactions with Akt kinases and phosphatases. In prostate cancer cells, caveolin-1 sustains Akt signaling by inhibiting the Akt phosphatases PP1 and PP2A through direct interaction with the CSD (Li et al., 2003). Growth factor stimulation by EGF or PDGF activates PDK1 in membrane raft nanodomains defined by Lyn localization that are spatially distinct from membrane regions of PTEN recruitment (Gao et al., 2011). This spatial segregation of activating kinase (PDK1) and negative regulation by phosphatases (PTEN) was proposed to be critical for Akt signaling, and disruption of this compartmentalization by ceramide impaired Akt activation (Goswami et al., 2005; Hajduch et al., 2008; Gao et al., 2011).

Flotillin nanodomains are a subset of membrane rafts distinct from caveolae that may also control RTK signaling. These structures are composed of oligomers of the highly conserved flotillin-1 and flotillin-2 proteins (Kurrle et al., 2012; Banning et al., 2014). While these nanodomains may also control PI3K/Akt signaling, much less is known about this phenomenon. Several studies that perturbed flotillin function observed impaired Akt signaling (Amaddii et al., 2012; Jang et al., 2015; Liu et al., 2015), yet it is not clearly established if this represents specific effects on PI3K-Akt signaling or broad regulation of RTKs. Consistent with the latter possibility, flotillins may function to control receptor membrane traffic such as that of IGF1R (Jang et al., 2015), or expression of specific receptor tyrosine kinases (Pust et al., 2013; Asp et al., 2014). Thus, both caveolae and flotillin nanodomains can contribute to control of Akt signaling by receptor tyrosine kinases at many levels, including at the level of the receptor thus broadly impacting many aspects of signaling. Much remains to be learned about the mechanism by which flotillins, caveolins and/or nanodomains formed by these proteins compartmentalize signals leading to Akt activation by receptor tyrosine kinases.

### Tetraspanin-Enriched Nanodomains

Tetraspanins are a large family of 33 proteins in humans, each with four membrane-spanning domains, that form membrane nanodomains through the interaction with other tetraspanins, integral membrane proteins, and cytoplasmic signaling proteins. Given the number of tetraspanin family members and their ubiquitous or tissue-specific distribution, it is no surprise that tetraspanins have been implicated in a diverse array of (patho)physiological processes and signaling pathways involved in immunity, angiogenesis, cancer, and many others (Yáñez-Mó et al., 2009; Charrin et al., 2014; Beckwith et al., 2015; Delos Santos et al., 2015; Berditchevski and Odintsova, 2016; Termini and Gillette, 2017; van Deventer et al., 2017; Schaper and van Spriel, 2018).

One of the primary functions of tetraspanins is the organization of plasma membrane receptors to facilitate signaling. Various tetraspanins, including CD9, CD63, CD81, CD82, and CD151 (Odintsova et al., 2000, 2003; Takahashi et al., 2007; Murayama et al., 2008; Devbhandari et al., 2011; Tugues et al., 2013; Berditchevski and Odintsova, 2016), have been reported to interact with specific RTKs. Early studies on tetraspanin CD82 demonstrated that ectopic CD82 expression in cells alters the plasma membrane distribution of EGFR (Odintsova et al., 2003). More recent studies using single particle approaches have revealed that CD82 confines EGFR in distinct regions of the plasma membrane, and loss of CD82 results in enhanced clathrin-mediated endocytosis of the receptor and impaired receptor signaling (Danglot et al., 2010).

Consistent with the ability of tetraspanins to control the dynamic nanoscale localization of RTKs, perturbations or alterations of tetraspanins impact certain aspects of RTK signaling, from the activity of receptors to specific signaling pathways. For instance, CD82 depletion or blocking antibody treatment attenuates Akt activation, resulting in induction of a pro-apoptotic phenotype (Nishioka et al., 2015). Furthermore, in endothelial cells lacking CD151, Akt activation is attenuated, resulting in impaired angiogenesis, which may be related to the requirement for tetraspanins for the activation of eNOS signaling (Takeda et al., 2007; Zheng and Liu, 2007). This regulation is complex, as perturbation of CD82 can also promote EGFR signaling to Akt activation in other contexts (Li et al., 2013).

Mechanistically, there remains much to be learned about how tetraspanin nanodomains control PI3K/Akt signaling. Tetraspanin domains are indeed enriched in specific signaling regulators such as certain PKC isoforms (Zhang et al., 2001), lipid enzymes such as phosphatidylinositol-4-kinase (Berditchevski et al., 1997; Yauch and Hemler, 2000; Carloni et al., 2004; Claas et al., 2005) and specific glycosphingolipids (Todeschini et al., 2007; Hakomori, 2010; Hakomori and Handa, 2016). The latter function in conjunction with CD82 to control signaling by EGFR and MET (Li et al., 2013). As with caveolin and flotillin nanodomains, it remains to be determined how tetraspanins may control RTK signaling leading to Akt activation, which could result either from specific recruitment and regulation of PI3K-Akt signaling intermediates or by broad control of RTKs controlling many signaling pathways. Consistent with the latter and with a context-dependent regulation of RTK signaling by tetraspanins, CD151 deletion attenuates ERK but not Akt activation in breast cancer epithelial cells expressing ErbB2 (Deng et al., 2012). It is plausible that the differences in Akt activation in response to CD151 perturbation are the result of cell-type specific effects, such as the expression of ErbB2, which is known to modulate the dependence of Akt activation on clathrin (Garay et al., 2015).

Future studies aimed at understanding how each of these can impact activation of PI3K/Akt signaling, either directly or in the context of complex regulation of signaling intermediates by compartmentalization within other nanodomains, will be very informative.

### Dorsal Ruffles (DRs) and Other Actin-Based Structures at the Plasma Membrane

Receptor tyrosine kinase signaling triggers dynamic remodeling of the cytoskeleton; in particular, activation of EGFR, PDGFR, IR, MET, and others leads to the rapid formation of actin-rich dorsal ruffles(DRs) at the plasma membrane (Abella et al., 2010; Hoon et al., 2012; Delos Santos et al., 2015; Yoshida et al., 2018). While not observed in all cells and contexts, DRs can serve to compartmentalize RTKs into spatially distinct membrane nanodomains for signaling. In addition, DRs act in parallel to RTK internalization by CME as a clathrin-independent mechanism for RTK internalization by macropinocytosis (Orth et al., 2006; Abella et al., 2010; Gu et al., 2011; Yoshida et al., 2018). Internalization of RTKs by DRs through the macropinocytic pathway requires Arp2/3-dependent actin polymerization and PI3K; disruption of each results in attenuated DR formation and RTK macropinocytosis (Dharmawardhane et al., 2000).

In cells in which they are observed, DRs also serve as a platform for PI3K/Akt signaling. In L6 myotubes, insulin stimulates recruitment of the Class I PI3K subunits p110α and p85 to DRs, leading to the production of PIP_3_ and subsequently, Akt1 recruitment to these structures (Khayat et al., 2000; Patel et al., 2003). Similarly, EGF stimulation of A431 cells leads to enrichment of PI45P_2_ and PIP_3_ in DRs, suggesting increased activity of class I PI3K in this compartment (Araki et al., 2007). In NIH3T3 cells, PIP_3_ production in DRs leads to the recruitment of the adaptor protein SH3YL1 along with SHIP2, which generates PI34P_2_; knockdown of either SH3YL1 or SHIP2 attenuated DR formation suggesting that PI3K/Akt signaling is essential for DR maturation (Hasegawa et al., 2011). Finally, it was recently shown that EGF and PDGF stimulate Akt activation in DRs, with distinct requirements for microtubules (Yoshida et al., 2018). Taken together, DRs comprise a distinct membrane-associated nanodomain that serves as a platform for Akt activation and signal termination via RTK macropinocytosis; this platform requires dynamic remodeling of the actin cytoskeleton and in certain contexts, microtubules.

### Akt Activation at the Primary Cilia

The primary cilia is a microtubule-based protrusion at the plasma membrane that acts as a hub for the integration of a number of important mechanochemical signals which regulate cellular growth, development, and quiescence. The primary cilia, of which there is typically only one per cell, is anchored via its assembly of microtubules to a centriolar anchor known as a basal body (Pan and Snell, 2007; Malicki and Johnson, 2017). The assembly of the primary cilia, known as ciliogenesis, occurs following mitosis largely in quiescent cells (Goto et al., 2017). Illustrating the importance of this structure, at least 35 human diseases (ciliopathies) are caused by mutations in genes involved in the function of cilia, including the primary cilia (Reiter and Leroux, 2017). Many signaling pathways are either activated in a localized manner at the primary cilia, or impact ciliogenesis, or both, including those triggered by Hedgehog, Notch, Wnt, Hippo, and certain GPCR ligands (Bangs and Anderson, 2017; Wheway et al., 2018). Here, we highlight studies that examined RTK-mediated Akt signaling in the primary cilia.

Platelet-derived growth factor (PDGF) receptor α is a RTK that localizes to the mature primary cilia, and following PDGF treatment, Akt is activated in the primary cilia of NIH 3T3 fibroblasts (Schneider et al., 2005). Genetic disruption of IFT88 or IFT20, two different component of the primary cilium intraflagellar transport system, attenuates or enhances PDGFRα and Akt activation at the ciliary basal body in fibroblasts, respectively, thus altering wound healing (Schneider et al., 2010; Clement et al., 2013; Schmid et al., 2018). As with caveolin and flotillin nanodomains, it remains to be determined if cilia specifically control PI3K-Akt signaling by localized recruitment of these signals to cilia, or by a broader regulation that occurs at the level of RTKs themselves, thus impacting many signaling pathways. New insight in the former possibility may obtained from observations that PI3KC2α is localized at the primary cilia basal body (Franco et al., 2014) and that PDGFRα stimulation of fibroblasts also leads to recruitment of phosphorylated Akt to the basal body, where it can phosphorylate its substrate Inversin to control ciliogenesis (Suizu et al., 2016).

### Ras Nanoclusters

The Ras proteins (H-Ras, K-Ras, and N-Ras) are a family of small GTPases that are important downstream effectors of RTK signaling (Schubbert et al., 2007; Simanshu et al., 2017). Following RTK activation by external stimuli, Ras proteins are recruited by Son of sevenless (SOS)-Grb2 to the tyrosine phosphorylated cytoplasmic residue of the receptor. From here Ras is activated by SOS GEF activity, leading to canonical activation of the Raf-MEK-ERK pathway. Single molecule and EM imaging revealed that Ras proteins form short-lived signaling nanodomains at the plasma membrane (Harding and Hancock, 2008; Abankwa et al., 2010; Janosi et al., 2012; Zhou and Hancock, 2015). These short-lived Ras nanodomains might regulate PI3K/Akt signaling independently of RTK-Grb2-Gab1 axis, as Ras proteins interact with the p110 catalytic subunit of Class I PI3K isoforms leading to its activation (Murthy et al., 2018), and genetic disruption of the Ras-PI3K interactions leads to impaired growth and development (Castellano and Downward, 2011). The Ras-PI3K p110α interaction is mediated by phosphorylation of K-Ras S191, and this causes formation of phosphorylated K-Ras nanoclusters that are distinct from the membrane population of non-phosphorylated K-Ras (Barceló et al., 2013). From here the Ras-PI3K interaction can stimulate the production of membrane PIP_3_ (Fukushima et al., 2018), which presumably triggers Akt activation. Consistent with control of PI3K-Akt signaling by Ras nanoclusters, a G12V mutant of K-Ras exhibits enhanced clustering of PI45P_2_ and an enhanced ability to elicit Akt activation compared to wild-type K-Ras (Zhou et al., 2017), but the mechanism by which this control occurs remains to be fully elucidated. Importantly, while these mechanisms of protein-protein interaction predicts that PI3K-Akt signaling can be triggered by Ras and thus controlled within Ras nanoclusters, this remains largely speculative and is in need of further investigation.

As discussed in the preceding sections, there are numerous mechanisms by which PI3K-Akt activation can depend on or be facilitated by plasma membrane nanodomains. That multiple such distinct nanodomains operate for different RTKs is perhaps not surprising given the diversity of signals and activation mechanisms of these receptors. However, there are also numerous examples of specific RTKs for which different studies have identified a role of distinct nanodomains in the activation of PI3K-Akt signals. For example, EGFR-dependent Akt activation requires clathrin, flotillin nanodomains and is also regulated by caveolin-1 and dorsal ruffles. This suggests several possibilities: (i) that several distinct nanodomains operate simultaneously to coordinate different specific molecular events in sequence that collectively lead to Akt activation or (ii) that distinct molecular and cellular contexts underlie unique requirements for specific nanodomains in specific situations. To this end, studies that extend beyond the establishment of a functional requirement for specific nanodomains to also resolve the specific molecular mechanisms by which nanodomains facilitate RTK signaling are very informative. Indeed, we previously showed that clathrin nanodomains are required for activation of PI3K-Akt signaling by EGFR only in the absence of ErbB2 (Garay et al., 2015), providing a molecular explanation for the context-dependent requirement for clathrin vs. other nanodomains for EGFR-dependent activation of PI3K signaling. Also, studies that undertake a systematic analysis of RTK signaling within nanodomains at the cell surface will further illuminate how each type of receptor and/or intracellular signal may engage and require distinct nanodomains either simultaneously or selectively, in a context-dependent manner. Such studies will reveal the molecular details of the nanoscale organization of the plasma membrane that is essential for PI3K-Akt signal activation.

## Localization of PI3K-AKt Signaling Among Endomembrane Compartments

A fundamental, yet still incompletely answered question is how is Akt able to synthesize diverse inputs into a response that targets only the appropriate substrates at the proper location. The conventional mechanism of Akt activation involves recruitment of inactive Akt to membrane sites of PI34P_2_ or PIP_3_ through interaction with its PH domain. Indeed, structural studies elucidated that binding of the Akt PH-domain to membrane phosphoinositides results in a conformational change that relieves the autoinhibitory interaction between the PH and catalytic domains, resulting in enhanced substrate binding capacity and Akt activation (Ebner et al., 2017a; Lučić et al., 2018). However, there are conflicting models regarding the ability of Akt to phosphorylate non-plasmalemmal substrates. The first model involves Akt activation on a particular membrane resulting in Akt becoming “locked” in an active conformation, which then allows redistribution of this active Akt to a multitude of other subcellular locales where it acts on its effectors (Meier et al., 1997; Calleja et al., 2009a,b; Antal and Newton, 2013). A second more recent model suggests that Akt is activated in a compartment-specific fashion, whereby inactive Akt is recruited to distinct subcellular locales for activation and is mostly unable to redistribute to other compartments once activated (Ebner et al., 2017a). Here, we first examine the evidence for plasma membrane activation of PI3K-Akt signals, then the evidence for activation of Akt on various specific endosomes, then lastly discuss the evidence for Akt activation in and/or redistribution to other compartments.

### Plasma Membrane Akt Activation

**FIGURE 1:**
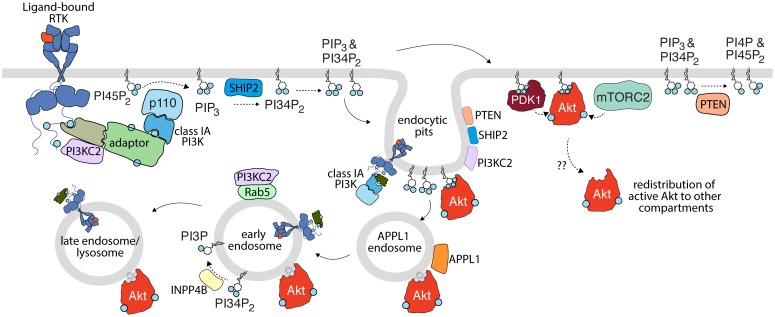
Activation of PI3K-Akt signaling by receptor tyrosine kinases. Upon activation, RTKs lead to recruitment and activation of class IA PI3K (with p110 catalytic subunit highlighted here), either by direct binding (not shown) or through various adaptors. This leads to the production of PIP_3_ from PI45P_2_; some PIP_3_ is subject to conversion to PI34P_2_ by SHIP2. PIP_3_ and PI34P_2_ lead to membrane recruitment and activation of PDK1, which together with mTORC2 phosphorylate Akt. Class II PI3K (PI3KC2) are also recruited to active RTK signaling complexes and are localized to clathrin endocytic pits, but the contribution of this class of PI3K to production of PI34P_2_ and/or Akt activation upon RTK activation is not clear. Turnover of PI34P_2_ and/or PIP_3_ by PTEN limits signaling to Akt. As discussed in detail in the text, spatial organization of signals in the plasma membrane, in nanodomains such as clathrin endocytic pits (e.g., PTEN, SHIP2, Akt, and PI3K) and specific endosomes (e.g., INPP4B, PI3KC2 in EEA1- and/or Rab5-early endosomes) further define compartment-specific Akt activation or negative regulation. The membrane traffic following RTK activation from the plasma membrane, including endocytosis and sequential transit through various endosomal compartments may be important for the delivery of plasma membrane-derived signals (such as specific phosphoinositides or even active RTKs themselves) for compartment-specific Akt activation. Akt may also redistribute from the site of activation to other compartments. While in some cases, Akt is shown to associate with specific lipids such as PI34P_2_ and PIP_3_ (shown in blue), Akt is depicted as bound to a gray lipid headgroup in some compartment or circumstances to indicate that the interaction of Akt with lipids is complex or unclear in such circumstances.

Consistent with the role of plasma membrane nanodomains in regulating Akt signaling, there is substantial evidence to implicate the plasma membrane as the primary site of Akt activation (Figure 1). Given that the substrate for the class I PI3K, PI45P_2_ is highly enriched at the plasma membrane (Di Paolo and De Camilli, 2006; Balla, 2013), it follows that PIP_3_ production occurs mainly within this compartment (Naguib, 2016). The recent development of sophisticated probes for lipid imaging revealed that PIP_3_ is indeed produced exclusively at the plasma membrane (Liu et al., 2018). Moreover, several studies identified that the vast majority of PI34P_2_ is generated by SHIP2-mediated dephosphorylation of PIP_3_ (Goulden et al., 2018; Liu et al., 2018), suggesting that PI34P_2_ is produced secondary to Class I PI3K, and as opposed to production by Class II PI3K (Figure 1). Importantly, two distinct probes identified either exclusive plasma membrane (Goulden et al., 2018) or plasma membrane and endosome (Liu et al., 2018) production of PI34P_2_. Notably, this localized production of PIP_3_ and PI34P_2_ was associated with isoform-specific localized activation of Akt, with Akt2 selectively activated at the plasma membrane or endosomal sites by PI34P_2_ while Akt1 and Akt3 display a preference for plasma membrane PIP_3_ (Liu et al., 2018). Thus, these findings support the model of activation of class I PI3Ks at the plasma membrane, leading to PIP_3_ production therein, which can then be coupled to direct activation of Akt at the plasma membrane (e.g., Akt1,3) or transport of PIP_3_ from the plasma membrane to endosomes, allowing for PI34P_2_ production and Akt2 activation in both compartments (Liu et al., 2018).

In addition to the roles of PI34P_2_ and PIP_3_ in Akt membrane recruitment and activation, other species of membrane lipids have also been implicated in driving the spatiotemporal organization of PI3K/Akt signaling at the plasma membrane. The PH domain of PDK1 binds PIP_3_ (Naguib, 2016), but it also displays a high affinity for phosphatidylserine (PS), and PI3K inhibition (and thus attenuation of PIP_3_ synthesis) fails to attenuate PDK1-PH domain association with the plasma membrane, while PS depletion results in accumulation of PDK1-PH in the cytoplasm (Lucas and Cho, 2011). Similarly, PS depletion renders the Akt-PH domain insensitive to stimulation by IGF1, resulting in its cytoplasmic accumulation (Huang et al., 2011). Akt activation is attenuated in fibroblasts expressing a mutant PDK1-PH domain that fails to associate with the membrane (Lucas and Cho, 2011), while mutation of the Akt-PH domain to yield a PS-binding defective mutant fails to activate Akt in response to IGF1 (Huang et al., 2011). In the latter study, the authors suggest that PS binding to Akt promotes a conformational change that facilitates PIP_3_ binding for subsequent T308 and S437 phosphorylation by PDK1 and mTORC2, respectively (Huang et al., 2011). PS is detected in the plasma membrane and on a variety of organelles (Fairn et al., 2011), thus suggesting that modulation of Akt activation by PS could occur at the plasma membrane as well as other compartments.

Insulin stimulation results in a PI3K-dependent accumulation of the Akt2 isoform at the plasma membrane in adipocytes, while Akt1 was localized to the cytoplasm (Sasaoka et al., 2004; Gonzalez and McGraw, 2009a). Akt1 was unable to elicit phosphorylation of the RabGAP AS160 to control membrane traffic of the facilitative glucose transporter GLUT4, while a mutant of Akt1 that promotes its plasma membrane accumulation rescued phosphorylation of AS160. The preferential recruitment of Akt2 to the plasma membrane following insulin stimulation may be partially mediated by ClipR-59, a CAP-gly domain-containing protein that directly interacts with phosphorylated Akt (Ding and Du, 2009). Furthermore, insulin-stimulated plasma membrane Akt recruitment was not only influenced by PIP_3_ production therein, but also controlled by ubiquitin-like protein 4A (Ubl4A), which by association with Arp2/3, generates actin structures that assist in delivering Akt to the plasma membrane for subsequent ligand-stimulated activation (Zhao et al., 2015). These studies support a model where Akt localization to the plasma membrane, either by PIP_3_ production or via delivery by other mechanisms, is critical for Akt activation.

The investigation of EGFR signaling supports further evidence for the plasma membrane-centric view of Akt activation. As previously mentioned in the section on clathrin nanodomains, interfering with clathrin (but not receptor endocytosis *per se*) attenuates Akt signaling, suggesting that the clathrin structures on the plasma membrane orchestrate Akt signaling (Garay et al., 2015). In cells depleted of all dynamin isoforms and thus with effectively completely arrested EGFR endocytosis, EGF stimulation leads to sustained, apparently normal Akt signaling (Sousa et al., 2012). In support of these findings, interfering with EGFR endocytosis or ubiquitination, both of which cause EGFR membrane retention, results in an upregulation of an EGF-dependent gene expression program, some of which is Akt-dependent (Brankatschk et al., 2012). Together, these studies suggest that plasma membrane EGFR signaling is sufficient to trigger normal Akt phosphorylation in the cell types examined, at least when assessing the overall activation of Akt and primary transcriptional outcomes.

### Activated Akt in Multiple Distinct Endosomes

Endocytosis of RTKs (EGFR, PDGFR, and others) by CME from the cell surface provides the cell with a means for regulating cell surface signaling while also directing signaling complexes to the various endomembrane compartments via fusion with the endosomal system (Figure 1). Throughout this process, endosomes are characterized by interactions with proteins involved in trafficking and sorting that facilitate the maturation of the nascent endosome. These proteins include members of the Rab GTPase family (e.g., Rab5, Rab7, and Rab11), APPL1, EEA1, and WDFY2 (Stenmark, 2009; Pálfy et al., 2012; Goldenring, 2015). Beyond merely providing a molecular signature to the endosomes that they occupy; these proteins are also involved in the spatiotemporal regulation of signaling proteins (including Akt) and lipids (including PI3P and PI34P_2_) that may originate at the endosomal level. As such, numerous studies have investigated the mechanisms of Akt signaling throughout the endosomal compartments that are traversed by signaling receptors after ligand binding.

Differences in the subcellular localization of the Akt isoforms have been documented, suggesting that Akt may be activated in a compartment-specific fashion. In general, activated Akt1 and Akt3 occupy similar compartments within the cell, including the plasma and nuclear membranes (Liu et al., 2018). Akt2, on the other hand, is consistently found in the cytoplasm, intracellular membranes, where it interacts with early endosomes, as demonstrated by colocalization with the early endosomal markers Rab5, APPL1, EEA1, and WDFY2 (Mitsuuchi et al., 1999; Walz et al., 2010; Li Chew et al., 2015; Liu et al., 2018). However, as noted above, some studies have also reported that Akt2 is selectively recruited to the plasma membrane upon insulin stimulation (Sasaoka et al., 2004; Gonzalez and McGraw, 2009a).

APPL1 demarks a subpopulation of early endosome through which some signaling receptors such as EGFR transit before eventually undergoing traffic to classical EEA1- and PI3P-positive early endosomes (Zoncu et al., 2009; Figure 1). Importantly, depletion of APPL1 attenuates Akt activation at this site (Schenck et al., 2008; Reis et al., 2015). APPL1 may contribute to sustained Akt activation in endosomes by facilitating interaction with the actin cytoskeleton, through the actin-binding protein MYO6 (Masters et al., 2017). Loss of MYO6 results in the shuttling of APPL1- and/or Rab5-positive endosomes from actin ruffles in the cell periphery to the perinuclear space, and impaired Akt activation in response to EGF stimulation. In addition, transition of cargo from PI3P-negative, APPL1-positive early endosomes to APPL1-negative, PI3P-positive early endosomes is regulated by Beclin-1, and as such signaling to Akt is controlled by Beclin-1, perhaps as a result of gating the duration of signaling from APPL1 early endosomes (Rohatgi et al., 2015). Thus, APPL1 might contribute to early endosomal Akt signaling by bringing inactive Akt into contact with early endosomes and delaying the transit of endosomes harboring active Akt through the endocytic pathway.

Subsequent to transit through APPL1 endosomes, some RTKs such as EGFR traffic to so-called classical early endosomes, demarked by EEA1, which associates with these compartments through PI3P binding by its FYVE domain (Murray et al., 2016; Figure 1). Both EEA1 and APPL1 are individually associated with Rab5-positive early endosomes, but they are typically found as distinct subpopulations (Masters et al., 2017), yet Akt can be detected in EEA1 endosomes in some circumstances (Nazarewicz et al., 2011). The Class II PI3K PI3KC2γ colocalizes with Rab5 positive endosomes (Braccini et al., 2015) suggesting a mechanism for localized production of PI34P_2_ at Rab5-positive early endosomes, which could lead to Akt activation therein. Indeed, loss of PI3KC2γ results in impaired Akt activation on endosomes. The inositol-3-phosphatase INPP4B is enriched in Rab5 positive early endosomes and loss of INPP4B results in enhanced Akt2 activation in this subpopulation of endosomes (Li Chew et al., 2015). This observation supports the model of localized PI34P_2_ production at early endosomes that is under the compartment-specific tight control of lipid phosphatases. Consistent with this model, Rab5 potentiates the activity of inositol-4-phosphatases, and loss of this endosomal phosphatase leads to enhanced PI34P_2_ levels (Shin et al., 2005).

In addition to early endosome subpopulations defined by the presence of APPL1 or EEA1, an additional, early endosomal population that lacks these markers but contains WDFY2 are essential for endocytosis of some receptor cargo (Hayakawa et al., 2006). WDFY2, through its FYVE domain, binds PI3P with high affinity and can interact with and co-localizes with Akt2, but not Akt1. Loss of WDFY2 results in loss of Akt2 protein levels and phosphorylation, which suggested a model in which initial activation of Akt2 at the plasma membrane leads to the protection of Akt2 protein from degradation by subsequently binding to WDFY2 endosomes (Walz et al., 2010).

Some RTKs transit from early endosomes to late endosomes *en route* to degradation in the lysosome (Sorkin and Goh, 2009; Goh and Sorkin, 2013; Critchley et al., 2018; Figure 1). The transition from early to late endosome to lysosome is characterized by the loss of Rab5 and recruitment of Rab7 (Rink et al., 2005); this switch in identity is also accompanied by changes in the phosphoinositide populations on the endosome. Early endosomes are abundant in PI3P, which is produced by the class III PI3K and Rab5 effector VPS34 (Jaber et al., 2016). PI3P is involved in the homotypic fusion of early endosomes, the recruitment of Rab7 GEFs, and components of the ESCRT complex. Collectively, the actions of many proteins facilitate the sorting and shuttling of the endosome along the endocytic pathway (Wallroth and Haucke, 2018), as well as degradation of material by incorporation of specific cargo such as RTKs into intralumenal vesicles destined for the lysosome (Piper and Katzmann, 2007; Bissig and Gruenberg, 2013).

The late endosome/lysosome has the potential to regulate Akt activation in two broad ways: (i) control of lysosomal sorting that impacts the rate of RTK degradation could modulate signals emanating from late endosomal receptors, thus affecting Akt signal duration, or (ii) the unique phosphoinositide profile of late endosomes/lysosomes may facilitate Akt activation separate from the plasma membrane or early endocytic compartments. Many instances of the former have been presented (Lemmon and Schlessinger, 2010; Goh and Sorkin, 2013). For example, alterations in EGFR membrane traffic that lead to accelerated lysosomal degradation result in shorter duration of receptor signaling, including that leading to Akt activation (Sigismund et al., 2008). The regulation of RTK degradation is sophisticated, as in some circumstances perturbation of lysosomal degradation by depletion of Rab7 surprisingly enhanced EGFR/HER2 proteasomal degradation and attenuated EGF-stimulated Akt phosphorylation (S473) (Wang et al., 2012). In this view, RTKs signals can remain active and highly relevant to sustain Akt activation at the late endosomes, resulting in enhanced pro-survival Akt signaling in cancer cells.

As previously mentioned, mTORC2 localizes to a subset of early (Rab5+) and late (Rab7+) endosomes in a PI3K-dependent manner (Ebner et al., 2017b). The PI3P-binding protein, Phafin-2, was identified as a positive regulator of EGFR degradation in response to EGF, by promoting EGFR shuttling through the endocytic/lysosomal degradation pathway (Pedersen et al., 2012). Induction of autophagy in cells triggers the lysosomal interaction between Phafin-2 and Akt, which leads to sustained Akt activation to control autophagy (Matsuda-Lennikov et al., 2014). Lysosomal mTORC2 leads to Akt activation in this compartment, thus suppressing chaperone-mediated autophagy (CMA) (Arias et al., 2015). Although it is unclear why the function of Akt activation on macro- or chaperone-mediated pathways differs, these studies suggest that late endosomes/lysosomes are sites of Akt activation.

### Localized Akt Activation in Distinct Compartments vs. Redistribution of Activated Akt

Given the requirement for PIP_3_ and PI34P_2_ for activation of Akt (as described above), a model of Akt activation initially emerged, which stipulated that Akt activation occurs at the plasma membrane or in endosomes enriched with PI34P_2_. Once activated in these specific compartments, this model proposes that Akt may adopt a “locked-active” conformation that permits redistribution of the active kinase to the nucleus (Meier et al., 1997) or other cellular compartments. This model was largely inferred by the vast number of Akt substrates and their diverse subcellular distributions. Additionally, the high rate of PIP_3_ turnover followed by sustained whole-cell Akt activation observed in early studies supported a model where transient PIP_3_ synthesis at the cell surface activated Akt for redistribution to the rest of the cell (Antal and Newton, 2013). Supporting this model, a FRET-based biosensor of Akt activity demonstrated that Akt activation in the plasma membrane preceded that in the cytosol, suggesting a redistribution of Akt once activated (Kunkel et al., 2005). Using a different FRET-based approach, Akt was found to form a complex with PDK1 prior to activation, such that upon ligand activation, the conformational change in Akt that occurs upon membrane binding allows phosphorylation of Akt, followed by dissociation from the membrane and redistribution of active Akt to distal sites (Calleja et al., 2007).

Several studies that revealed that Akt activation by RTKs occurs primarily at the cell surface and does not outright require receptor endocytosis *per se* (Brankatschk et al., 2012; Sousa et al., 2012; Garay et al., 2015) are broadly consistent with a model in which Akt is activated at the plasma membrane and then redistributes to other locales. However, directly studying active Akt redistribution is technically challenging, and only a small number of studies have directly examined this “locked-active” Akt redistribution model. In endothelial cells, internalization of the complement membrane attack complex (MAC) is mediated by CME and Rab5, leading to recruitment of activated Akt to MAC-containing endosomes. In these cells, inhibition of CME by clathrin siRNA had no effect on Akt phosphorylation, suggesting that in this case, Akt is activated at the membrane, independently of clathrin, from where it is then redistributed (Jane-wit et al., 2015).

The redistribution of “locked-active” Akt may occur via microtubules. Active Akt can associate with microtubules through interaction with the microtubule binding protein dynactin p150 (Jo et al., 2014). Inhibiting microtubule polymerization with nocodazole did not inhibit initial Akt activation by IGF1 stimulation; however, nocodazole treatment attenuated Akt signaling after the initial IGF1 stimulus. This suggests that microtubule polymerization possibly acts as a mechanism for redistributing active Akt to other parts of the cell to sustain Akt signaling.

Compared to the well-established mechanisms for Akt activation at the plasma membrane and in a subset of endosomes, much remains to be understood about the mechanisms that gate Akt activation that results in localized Akt activity in other membrane compartments. An important recent study found that while PI34P_2_ or PIP_3_ binding to the Akt PH-domain allosterically activates this kinase, the activity of Akt rapidly returns to basal upon dissociation from PIP_3_ or PI34P_2_ (Ebner et al., 2017a). This study thus challenges the “locked-active” Akt model in which the kinase is capable of redistribution from the plasma membrane to other compartments. Instead, the findings of this study suggest that Akt activity is restricted to specific membrane compartments in which it is initially activated, as dissociation of Akt from lipid binding would render Akt inactive. This model suggests instead that Akt is only activated in compartments enriched with compatible lipid ligands, such as PI34P_2_ and PIP_3_.

While the evidence that Akt requires ongoing association with membranes to sustain activity is thus strong, it is less well understood how certain compartments in which Akt has been reported, such as the late endosome or lysosome, could support Akt activation, due to the paucity of information of enrichment or production of PIP_3_ or PI34P_2_ in these compartments. Further studies have implied that Akt can be activated at other distinct subcellular locales, including the nucleus (Wang and Brattain, 2006; Santi and Lee, 2010), mitochondria (Santi and Lee, 2010), and the endoplasmic reticulum (Betz et al., 2013), again begging the question of how PIP_3_ or PI34P_2_ can be produced or enriched in these compartments to support Akt activation. Indeed the Class II PI3K PIK3C2β is recruited to the lysosome to produce PI34P_2_, but this did not appear to regulate Akt and instead suppressed mTORC1 signaling (Marat et al., 2017). It is possible that additional mechanisms such as chaperones could support redistribution of Akt among compartments once activated. Future studies that can glean further insight into the mechanisms of localized Akt activation in specific compartments, and/or in possible modes of redistribution of active Akt will be very informative.

## Compartment-Specific AKt Functions and Outcomes

Given the sheer number of Akt substrates that have been described in the literature, it is no surprise that Akt exhibits specific effects in different subcellular locales. A detailed analysis of the effects of Akt on each substrate, in each subcellular compartment is beyond the scope of this review, which can instead be found in several recent reviews (Manning and Cantley, 2007; Manning and Toker, 2017). Instead, the following section highlights some key regulatory functions of Akt in distinct organelles and how dysregulated Akt activity in these locations can contribute to disease. A particular emphasis is placed on how localized Akt activity coordinates the activity of key signaling nodes in the regulation of cellular energy metabolism for regulation of cell growth and apoptosis.

### Plasma Membrane Akt May Support Formation of Invadopodia for Cancer Cell Metastasis

**FIGURE 2:**
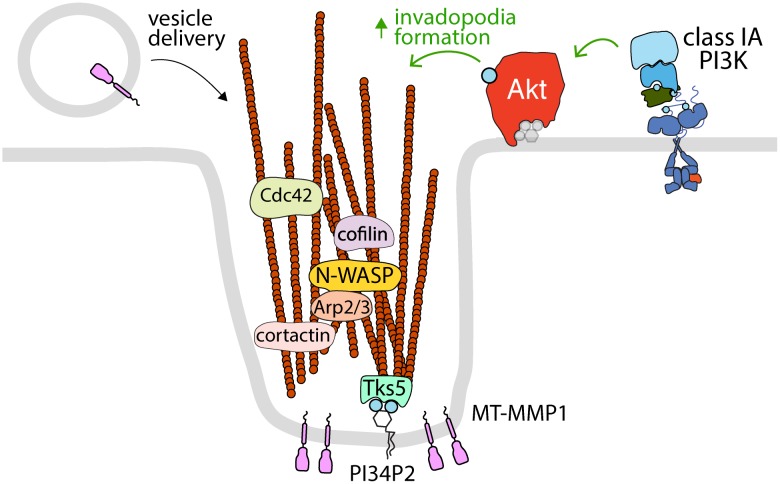
Akt controls the formation of invadopodia at the plasma membrane. The activation of specific RTKs leads to the formation of invadopodia, which requires class IA PI3K and Akt. Shown is the control of various proteins that control actin polymerization and dynamics (cortactin, cofilin, N-WASP, Arp2/3, and Cdc42) that are localized to and/or required for Akt-dependent invadopodia formation at the plasma membrane. Also shown are the PI34P_2_-binding protein Tks5, which links actin filaments to the plasma membrane in invadopodia, and MT-MMP1, which is delivered to invadopodia by vesicle carriers and serves to degrade extracellular matrix. Since invadopodia are formed at the plasma membrane, it follows that these structures are likely dependent on plasma membrane Akt pools.

Invadopodia are actin-rich, plasma membrane-associated structures that play a crucial role in remodeling of the extracellular matrix (ECM) for cell migration and invasion. The formation of invadopodia is particularly relevant in cancer metastasis, whereby degradation of the ECM by invadopodia-associated matrix metalloproteases (MMPs) facilitates dissemination of cancer cells to other compartments (Hoshino et al., 2013; Eddy et al., 2017; Figure 2). Invadopodia formation occurs in stages and is often triggered by RTK signaling; stimulation with EGF, VEGF, PDGF, and other RTK ligands can promote the initiation of invadopodia (Hoshino et al., 2013). Following RTK activation, invadopodia initiation requires recruitment of the regulators of actin polymerization, N-WASP, Arp2/3, cofilin, to the actin-cortactin complex (Hoshino et al., 2013; Eddy et al., 2017). This facilitates actin polymerization, which is then anchored to the plasma membrane by interaction with the PI34P_2_-binding scaffold protein Tks5 (Murphy and Courtneidge, 2011). From here, the invadopodia mature via additional Cdc42 (or other Rho-family GTPases)-mediated actin polymerization and recruitment of Membrane Type-1 Matrix Metalloproteinase (MT1-MMP), which leads to rapid degradation of the underlying ECM (Hoshino et al., 2013; Castro-Castro et al., 2016; Eddy et al., 2017).

Phosphoinositides play a critical role in the regulation of invadopodia formation and activity, at least in part due to the regulation of Akt activation (Figure 2). The balance between formation of PIP_3_ and PI34P_2_ by Class I PI3Ks and phosphatases appears to modulate the metastatic potential of many cancer cell lines (Fukumoto et al., 2017; Malek et al., 2017). In MDA-MB-231 breast cancer cells, knockdown of the Class IA PI3K p110α catalytic subunit or chemical inhibition of class I PI3K attenuates invadopodia formation and Akt activation, while activating mutations of PIK3CA (which encodes p110α) promote invadopodia formation and Akt activation (Yamaguchi et al., 2011). The control of invadopodia formation requires Akt downstream of Class I PI3K activity, as knockdown of either PDK1 or Akt recapitulated the effects of p110α deletion (Yamaguchi et al., 2011). Interestingly, expression of a chimeric Akt that is constitutively active but lacks targeting specific to invadopodia (myristoylated Akt) attenuated invadopodia formation, suggesting that localized Akt at the site of invadopodia initiation and not general Akt activation on cellular membranes is required for invadopodia formation (Yamaguchi et al., 2011). Also in MDA-MB-231 breast cancer cells, knockdown of SHIP2 and PTEN differentially impacted invadopodia formation (Fukumoto et al., 2017). This yet again links PI3K/Akt signaling to invadopodia formation. Similarly, loss of PTEN or the 4-phosphatase INPP4B results in accumulation of PI34P_2_ at the plasma membrane in MCF10a breast cancer cells and loss of both PTEN and INPP4B substantially enhanced Akt activity and invadopodia formation (Malek et al., 2017). While a role for a plasma membrane-specific pool of Akt in the control of invadopodia formation and dynamics remain to be thoroughly investigated, given that invadopodia are protrusions of the plasma membrane, it is perhaps expected that these structures are controlled by plasma membrane pools of Akt.

### Akt Control of GSK3 at Early Endosomes

GSK3 is a ubiquitously expressed serine/threonine kinase that was first identified as a regulator of glycogen synthase activity. Similar to Akt, GSK3 acts on over 100 known substrates with unique tissue and subcellular distributions, and as such, it plays an important role in regulating a diverse array of cellular processes. One of the overarching themes of GSK3-mediated regulation is the concept that upon phosphorylation by GSK3, many substrates are inactivated or targeted for degradation (Chiara and Rasola, 2013; Beurel et al., 2015; Manning and Toker, 2017; Bautista et al., 2018). GSK3 is negatively regulated by phosphorylation at S21 and S9, found within a conserved motif in GSK3α and GSK3β, respectively.

**FIGURE 3:**
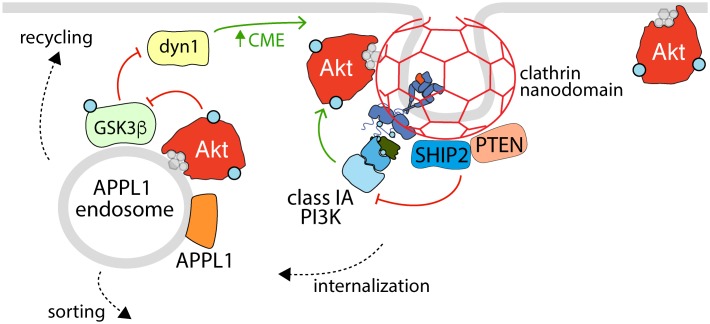
The reciprocal regulation of Akt activation, endocytosis, and APPL1 endosomes. Shown is an active RTK within a clathrin nanodomain (some of which lead to production of clathrin-coated vesicles for receptor internalization), with associated active class 1A PI3K (e.g., linked via phosphorylated Gab1 to EGFR, as in Garay et al., 2015). Also enriched within clathrin nanodomains are the phosphatases SHIP2 and PTEN, which control clathrin nanodomain dynamics and may thus control PI3K-Akt signaling, and Akt itself. Following internalization, some RTKs and associated signals are delivered to APPL1 endosomes, which also harbor active Akt that serves to phosphorylate (and inactivate) GSK3ß, a phenomenon which relieves the inhibition of GSK3ß on dynamin-1, which in turn regulates formation of clathrin structures and endocytosis at the cell surface. Hence, the activation of Akt at the plasma membrane and within APPL1 endosomes both requires clathrin structures at the cell surface and promotes formation of clathrin structures for internalization, establishing a positive feedback loop to amplify PI3K-Akt signal propagation downstream of certain RTKs.

Akt is one of a number of regulatory kinases that can phosphorylate GSK3 in response to stimuli, and there is evidence to suggest that this event occurs at the level of early endosomes, which provides regulatory feedback cues to components of the endocytic pathway (Figure 3). GSK3 phosphorylation occurs in a unique subset of endosomes that are APPL1 positive and TSC2 negative, and this requires APPL1 and PI3K activity (Schenck et al., 2008; Reis et al., 2015). The phosphorylation of GSK3 by Akt is mediated by the Akt2 isoform at early endosomes (Braccini et al., 2015). Notably, inhibition of CME, which prevents PI34P_2_ delivery to early endosomes, attenuates GSK3β phosphorylation but not that of FoxO or S6K, suggesting that early endosomes are a critical site for GSK3β regulation but perhaps not that of other Akt substrates (Liu et al., 2018). Accordingly, disrupting CME-derived membrane traffic results in accumulation of phosphorylated Akt in APPL1 endosomes and enhanced GSK3 phosphorylation, which leads to reduced dynamin-1 phosphorylation that promotes clathrin assembly (Reis et al., 2015). As such, endosomal Akt-GSK3 signaling may function as part of a positive feedback loop with clathrin-dependent Akt activation (Garay et al., 2015; Schmid, 2017) to potentiate Akt signaling.

### Akt Regulation of TSC2 and Other Processes at Lysosomes

Several lines of evidence suggest that Akt has compartment-specific functions that involve localization to the lysosome. As the site of mTORC1 activation, the lysosome is at the confluence of pathways that integrate nutrient and growth factor signaling for the control of cell growth. Activation of mTORC1 at the lysosome is accomplished through the combined effects of two separate, yet equally important pathways that converge upon mTORC1 to facilitate its full activation. In the first pathway, Rag GTPases are stimulated in the presence of amino acids and form a heterodimer that facilitates recruitment of mTORC1 to the lysosomal surface through interaction with Raptor (Demetriades et al., 2014; Lim and Zoncu, 2016; Sabatini, 2017; Saxton and Sabatini, 2017; Wolfson and Sabatini, 2017). From here, mTORC1 is activated by PI3K-Akt signaling which impacts the activation of Rheb, a second class of GTPase (Tee et al., 2003; Long et al., 2005). In the absence of mitogenic signaling, Rheb activity is suppressed by the GAP activity of the TSC complex, which is comprised of TSC1, TSC2, and TBC1D17. Mitogenic signaling impairs TSC-mediated suppression of Rheb, thus leading to activation of mTORC1 (Inoki et al., 2002, 2003; Tee et al., 2003; Long et al., 2005).

**FIGURE 4:**
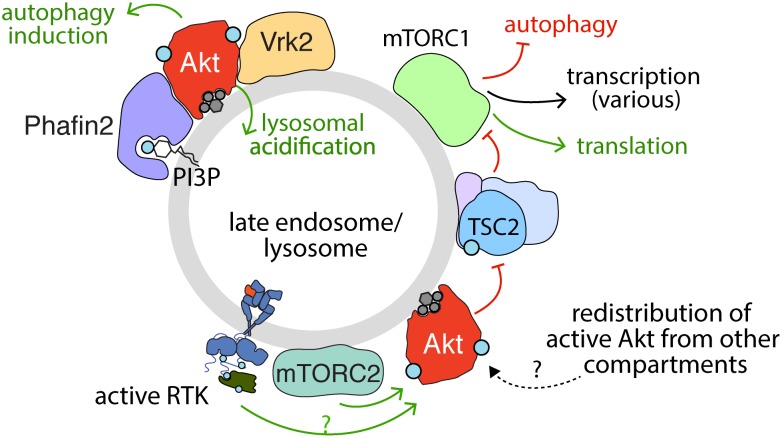
Activation, recruitment and function of Akt at the late endosome/lysosome. Sustained signaling by RTKs and membrane traffic at the late endosome/lysosome are required for sustained activation of Akt downstream of some RTKs. While the complete mechanism of Akt activation specifically at the lysosome remains to be elucidated, evidence for the localized activation of Akt at lysosomes is provided by the localization of mTORC2 therein. Akt may also be recruited to the lysosome following activation in another compartment. The PI3P binding protein Phafin2 and the serine/threonine kinase Vrk2 serve to recruit Akt to the lysosome, required for control of Akt-dependent lysosomal acidification and autophagy induction. In addition, given the localization of TSC2 (part of TSC complex) to the late endosome/lysosome, the phosphorylation of TSC2 by Akt may also occur in this compartment. Akt phosphorylation of TSC2 causes dissociation from the lysosome, which relieves the inhibition on mTORC1, allowing activation of mTORC1, which in turn impacts many cellular functions, of which some selected examples are shown.

Specifically, the activation of Akt by many RTKs elicits phosphorylation of TSC2, resulting in its inactivation and dissociation from the lysosome (Menon et al., 2014), thus allowing Rheb-dependent mTORC1 activation (Figure 4). The highly contextualized nature of mTORC1 activation at the lysosome by growth factor signaling through Akt and nutrient availability ensures that mTORC1 is only activated when the cellular microenvironment favors growth. Given the localization of TSC2 to the lysosome, Akt recruitment to or activation at the lysosome would seem to be required for phosphorylation of TSC2 and thus activation of mTORC1.

This compartment-specific role of Akt activation is consistent with the activation of Akt at the lysosome proposed to contribute to the regulation of CMA (Arias et al., 2015; Hirata et al., 2018; Figure 4). Indeed, lysosomal Akt promotes autophagy through interactions with lysosomal proteins such as Phafin2 and the serine/threonine kinase VRK2 (Matsuda-Lennikov et al., 2014; Hirata et al., 2018). Lysosomal Akt and VRK2 promote lysosomal acidification and silencing VRK2 by siRNA attenuates lysosomal Akt activation (Hirata et al., 2018). While many factors such as ubiquitinylation also control Akt turnover, such as by the proteasome or by caspases (Liao and Hung, 2010), these studies highlight that Akt also thus exerts control over global protein stability, likely as a result of lysosome-specific functions. Hence, multiple lines of evidence indicate lysosomal-specific functions of Akt.

### Nuclear Akt Promotes Cancer Growth and Chemotherapeutic Resistance

Upon stimulation of some RTKs, active Akt is detected in the nucleus (Wang and Brattain, 2006; Santi and Lee, 2010), and this can be delayed ∼30 min subsequent to initial Akt activation detected at the plasma membrane (Meier et al., 1997). Active nuclear Akt in the nucleus is protected from nuclear export and degradation by interaction with B23/NPM in the nucleus, a phenomenon which has a net effect of promoting cell cycle progression (Lee et al., 2008). Akt activity in the nucleus has been best described in the context of regulating nuclear/cytoplasmic localization of the Forkhead Box O Family (FoxO) members, a set of highly conserved transcription factors that control apoptosis, cell division, and metabolism (Manning and Toker, 2017). Akt regulates FoxO localization by directly phosphorylating three conserved regions on the FoxO proteins, which results in their association with 14-3-3 and retention in the cytoplasm (Brunet et al., 1999). As a result of FoxO cytoplasmic retention, nuclear Akt activity attenuates expression of the FoxO gene expression program, including genes involved in promoting apoptosis (FasL, Bim) and cyclin-dependent kinase inhibitors (p27) (Brunet et al., 1999; Trotman et al., 2006; Zhang et al., 2011). Thus, the net effect of active Akt in the nucleus is suppression of FoxO-mediated expression of genes that promote apoptosis and inhibit growth, and thus enhanced cell cycle progression and cell survival.

This role of nuclear Akt has important implications for cancer treatment. Akt activating mutations (in genes such as PIK3CA, PIK3CB, PIK3R1, PTEN, AKT, TSC1/2, and mTOR) are common in many tumors and are viewed as attractive therapeutic targets (Okkenhaug et al., 2016; Janku et al., 2018). However, prolonged Akt inhibition in cancer cell lines leads to FoxO-mediated upregulation of the RTK oncogenes Her3, IGF-1R, and the insulin receptor, possibly through relief of the negative feedback associated with RTK signaling (Chandarlapaty et al., 2011). In lung cancer xenografts, Akt inhibition alone fails to prevent tumor growth, and this is correlated with enhanced RTK activity. Interestingly, the sensitivity to Akt inhibitors is improved with adjuvant administration of EGFR/ErbB2 inhibitors, suggesting that targeting different components of the EGFR/PI3K/Akt signaling axis can overcome the compensatory RTK activity that accompanies inhibition of Akt alone (Chandarlapaty et al., 2011).

Akt is also active in the nucleus in response to various forms of DNA damage (Szymonowicz et al., 2018). Doxorubicin and other chemotherapeutic agents as well as ionizing radiation exert their cytotoxic effects, in part, by inducing DNA damage and subsequent cell cycle arrest and apoptosis (O’Connor, 2015). These effects are countered by repair pathways that respond to DNA damage, including activation of Akt by DNA-dependent protein kinases (DNA-PKs), which collectively activate various DNA repair pathways (Feng et al., 2004; Park et al., 2009; Liu et al., 2014; Toulany et al., 2017; Szymonowicz et al., 2018). Further studies that contribute to the understanding of how the nuclear functions of Akt activated by DNA damage may interface with Akt activated by RTK signaling will be valuable, given that active nuclear Akt is a central driver of cancer signaling and chemotherapeutic resistance and represents an attractive target for cancer therapy (Adini et al., 2003; Hui et al., 2008).

### Mitochondrial Akt Coordinates Energy Metabolism and Apoptosis

**FIGURE 5:**
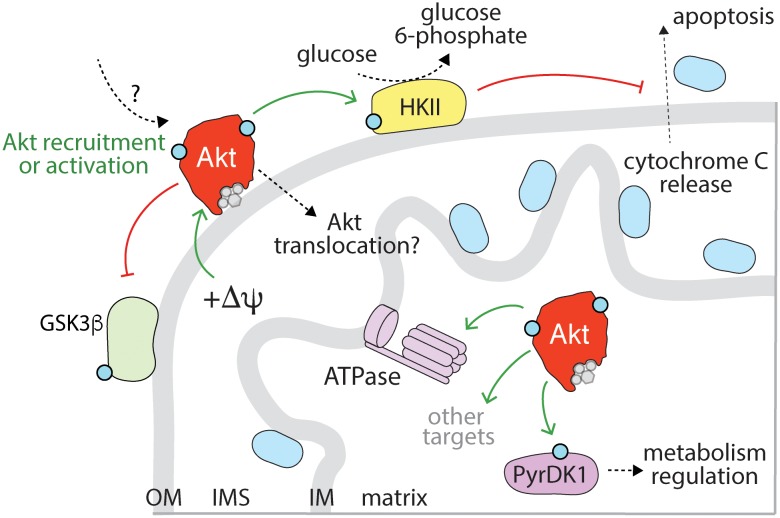
Mitochondrial Akt controls apoptosis and metabolism. Akt is recruited to the mitochondria upon activation of specific RTKs, in a manner that requires mitochondrial membrane potential (+ΔΨ). Mitochondrial Akt phosphorylates a number of substrates within this compartment, including GSK3ß and hexokinase II (HKII). Akt phosphorylation of HKII leads to enhanced activity, retention of HKII at the mitochondria and suppression of cytochrome c release (which would otherwise lead to apoptosis). Akt may also phosphorylate the mitochondrial ATPase. A wide range of mitochondrial substrates are controlled by mitochondrial Akt following hypoxia, including pyruvate dehydrogenase kinase I (PyrDK1). While activation of some RTKs may also lead to Akt localization within mitochondria, this mechanism requires further examination. OM, outer membrane; IMS, intermembrane space; IM, inner membrane.

Nutrients and growth factors trigger Akt mobilization to the mitochondria where it plays a central role in regulating cellular energy metabolism and apoptosis (Santi and Lee, 2010; Betz et al., 2013; Figure 5). This role of Akt is particularly important in cancer cell signaling, where dysregulated mitochondrial energy metabolism and apoptosis have been implicated in promoting tumor growth (Wallace, 2012). Mitochondrial signaling is required for the localized activation or redistribution of Akt to mitochondria, as disruption of mitochondrial membrane potential attenuates IGF1-stimulated Akt activation in the mitochondria (Bijur and Jope, 2003). Furthermore, disruption of mTORC2 signaling by Rictor deletion attenuates Akt activation at mitochondrial-associated ER membranes (Betz et al., 2013).

Accumulation of active Akt in the mitochondria following growth factor stimulation leads to the phosphorylation of a number of substrates responsible for energy metabolism, including hexokinase-2 (HK2), ATP-synthase, and GSKβ3 (Gottlob et al., 2001; Bijur and Jope, 2003; Beurel and Jope, 2006; Miyamoto et al., 2008; Roberts et al., 2013; Chae et al., 2016; Figure 5). Further, hypoxia induced activation of mitochondrial Akt, which in turn led to phosphorylation of a wide range of mitochondrial substrates including pyruvate dehydrogenase kinase I (Chae et al., 2016). It remains to be determined to what extent the hypoxia-induced Akt program is recapitulated by RTK activation of Akt. Moreover, while translocation of Akt to mitochondria upon insulin and IGF1 stimulation has been reported (Bijur and Jope, 2003), how Akt activated outside of the mitochondria may translocate into mitochondria is unclear, as are possible mechanisms by which RTK-derived signals may trigger activation of Akt within the mitochondria. The net effect of these individual phosphorylation events elicited by Akt at the mitochondria is a shift in cellular metabolism toward glycolytic pathways (Roberts et al., 2013; Chae et al., 2016), reduction in oxidative damage and suppression of apoptosis (Chae et al., 2016). The effects of mitochondrial Akt on HK2 are central to the coupling of energy metabolism and apoptosis; as the first committed step in glycolysis, HK2 phosphorylates glucose to glucose-6-phosphate (G6P). G6P negatively regulates HK2, resulting in its dissociation from the mitochondria. However, HK2 is stabilized on the mitochondria by Akt phosphorylation at T473 (Roberts et al., 2013), thus potentiating glycolytic metabolism.

Akt-mediated phosphorylation of HK2 also attenuates apoptosis by preventing cytochrome c release from the mitochondria (Gottlob et al., 2001; Miyamoto et al., 2008). The anti-apoptotic effects of Akt/HK2 on promoting mitochondrial integrity represents a separate arm of the intrinsic apoptosis pathway, as dissociation of Akt/HK2 from the mitochondrial membrane is sufficient to induce apoptosis in the absence of Bax/Bak (Majewski et al., 2004). Mitochondrial Akt also inhibits apoptosis by blocking mitochondrial caspase-3 activation, an effect specific to mitochondrial Akt, as overexpression of a constitutively active, mitochondrial-targeted Akt blocked caspase activation in the presence of PI3K inhibitors (Su et al., 2012). Thus, the concerted effects of active Akt at the mitochondria promotes glycolysis and inhibits the intrinsic apoptosis pathway, such that in cancer, these features promote cell growth and survival.

## Conclusion and Perspectives

The many and diverse substrates of Akt require tight control and regulation of this important kinase. This regulation is afforded in part by the intricate orchestration of Akt activation and function in space and time in many different endomembrane compartments, including the plasma membrane, endosomal compartments, mitochondria and the nucleus. This compartmentalization of Akt functions provides an important mechanism to allow context-specific outcomes of Akt activation. While this is an attractive model, several important aspects of this spatial compartmentalization of Akt activation and function are still poorly understood.

There is indirect evidence of Akt activation on some internal membranes, such as from perturbations of late endosome/lysosomal traffic that disrupt Akt activation, and the possible activation of Akt on or in mitochondria. However, there is little direct evidence of PIP_3_ and PI34P_2_ production on some of these internal membrane compartments that are proposed to support Akt activation. For the case of lysosomes, the localization of mTORC2 to this compartment is indeed consistent with localized activation of Akt, yet the strict requirement of Akt activation on PIP_3_ or PI34P_2_ indicate that our understanding of Akt activation at the lysosome and other internal organelles remains incomplete. Interestingly, while PI3KC2 is present at the lysosome, this lysosome-localized lipid kinase did not appear to contribute to Akt activation (Marat et al., 2017). Resolving how PIP_3_ and/or PI34P_2_ can be produced in any cellular compartment other than the plasma membrane and early endosomes as part of canonical Akt activation will be very informative. Indeed, the development and use of novel lipid biosensors as recently reported for PI34P_2_ (Goulden et al., 2018; Liu et al., 2018) or resolving other aspects of the mechanism of activation of Akt specific to various compartments will be very informative.

Several lines of evidence suggest that endocytic portals, likely to be clathrin-coated structures and vesicles, are important points of spatial convergence of many aspects of PI3K-Akt signaling. Plasma membrane clathrin structures are sites of signaling that lead to PI3K activation (Delos Santos et al., 2015, 2017; Garay et al., 2015), and Akt itself is enriched in these structures (Rosselli-Murai et al., 2018). In addition to these signals that positively regulate PI3K-Akt activation, clathrin structures are also enriched in lipid phosphatases including PTEN (Rosselli-Murai et al., 2018) and SHIP2 (Nakatsu et al., 2010), thus making clathrin nanodomains an important platform for spatial coordination of both positive and negative regulation of PI3K activation. Notably, the accumulation of PI34P_2_ on internal membranes leading to activation of Akt2 required both SHIP2 and internalization (perhaps of PIP_3_) from the plasma membrane (Liu et al., 2018). This model proposes that early endosome PI34P_2_ is thus derived from plasma membrane PIP_3_. While this is an interesting model, this is difficult to reconcile with the well-established recruitment of many lipid phosphatases within clathrin-coated structures and vesicles, and the very rapid turnover of plasma membrane phosphoinositides in vesicles upon scission from the plasma membrane (Krauß and Haucke, 2007; Zoncu et al., 2009; He et al., 2017). Hence, plasma membrane clathrin nanodomains and endocytic vesicles may indeed be spatial bottlenecks that both allow PI3K-Akt activation in coordination with negative regulation of this signaling axis to prevent aberrant signaling. However, much remains to be determined about how PI3K-Akt signals are coordinated in plasma membrane clathrin nanodomain platforms and endocytic vesicles derived therefrom.

In addition, many of the functions of Akt that are thought to be compartment-specific are inferred, largely from the localization of Akt to specific compartments that each harbor unique substrate pools. The development of methods to specifically perturb or alter Akt in a compartment specific manner (Maiuri et al., 2010) will be instrumental in resolving how compartment-specific activation or redistribution of Akt impacts cell physiology. With the important roles that Akt plays in the context of normal human health and development, as well as the mounting evidence for a critical role of disruption of Akt function in diseases such as cancer and type II diabetes, understanding the molecular mechanisms that underlie the compartment-specific activation and functions of Akt over the coming years will have substantial impact.

## Author Contributions

MS, GF, and CA wrote and edited this review manuscript.

## Conflict of Interest Statement

The authors declare that the research was conducted in the absence of any commercial or financial relationships that could be construed as a potential conflict of interest.
